# Outage Probability and Ergodic Capacity of a Two-User NOMA Relaying System with an Energy Harvesting Full-Duplex Relay and Its Interference at the Near User

**DOI:** 10.3390/s20226472

**Published:** 2020-11-12

**Authors:** Hoang Van Toan, Tran Manh Hoang, Tran Trung Duy, Le The Dung

**Affiliations:** 1 Faculty of Telecommunications Services, Telecommunications University, Khanh Hoa 650000, Vietnam; hoangvantoan@tcu.edu.vn (H.V.T.); tranmanhhoang@tcu.edu.vn (T.M.H.); 2 Department of Telecommunications, Posts and Telecommunications Institute of Technology, Ho Chi Minh 700000, Vietnam; trantrungduy@ptithcm.edu.vn; 3 Division of Computational Physics, Institute for Computational Science, Ton Duc Thang University, Ho Chi Minh 700000, Vietnam; 4 Faculty of Electrical and Electronics Engineering, Ton Duc Thang University, Ho Chi Minh 700000, Vietnam

**Keywords:** NOMA, energy harvesting, full-duplex, inter-user interference, outage probability, ergodic capacity

## Abstract

In this paper, we consider a two-user downlink full-duplex (FD) non-orthogonal multiple access (NOMA) relay system where the FD relay uses an energy harvesting (EH) technique to assist the communication between the base station and far user over flat, independent and non-identically Rayleigh fading channels. Importantly, since the relay operates in FD mode, we take into account the effect of the interference caused by relay on the near user. Considering this EH-FD-NOMA relay system, we derive the exact mathematical expressions of the outage probabilities and ergodic capacities of near and far users. Monte–Carlo simulations verify the accuracy of our analytical method. Numerical results provided in this paper allow system designers to clearly see not only the impacts of the power distribution factor and the self-interference cancellation capacity of the relay but also the influence of the strength of inter-user interference at the near user on the outage performances and ergodic capacities of both users.

## 1. Introduction

The rapid development of mobile information systems and the Internet of Things (IoT) sets new requirements and challenges for the fifth-generation (5G) wireless communications [1]. The performance requirements that a 5G radio system needs to achieve compared with a 4G radio system are very high: spectrum efficiency increases from 5 to 15 times; the number of connections is very large numbers, can be dozens of times higher, at least 10^6^ connections per km^2^ with small latency (less than 1ms) and can support various radio services [2].

Consequently, the non-orthogonal multiple access (NOMA) technique was proposed to meet the demand for increasing user connections in 5G wireless systems. The main idea of NOMA is to support non-orthogonal resource allocation among users. It can be classified into two main categories: power-domain NOMA [3] and code-domain NOMA [4]. For the power-domain NOMA, according to the channel quality, the different power level is assigned for each user simultaneously and on the same frequency to generate superposition coded symbol at the transmitter side. At the receiver side, thanks to the difference, the signal intended for each user can be decoded by using the successive interference cancellation (SIC) technique. In contrast, code-domain NOMA is similar to code division multiple access (CDMA) or multi-carrier CDMA. The main difference compared to CDMA is that the spreading sequences are limited to sparse sequences or non-orthogonal low cross-correlation sequences in code-domain NOMA [5]. Many researchers have demonstrated that power-domain NOMA can be used effectively to meet requirements of 5G technologies and can further enhance the performance of other wireless technologies, such as multiple-input multiple-output (MIMO) [6], cooperative [7], millimeter wave [8], cognitive radio (CR) [9], and energy harvesting (EH) [10]. Therefore, we also consider power-domain NOMA in this paper.

Besides, full-duplex (FD) operation has recently attracted significant attention due to its capacity to double the spectral efficiency compared to traditional half-duplex (HD) relaying. However, the practical throughput gain of FD operation is limited by the self-interference (SI). Fortunately, leveraging recent advances in antenna and transceiver design helps to cancel the SI up to the receiver noise floor [11], that making FD a promising solution for implementing future wireless system.

Mainly, the combination of NOMA and FD relaying has emerged as a promising solution to meet the high spectral efficiency requirements of 5G radio systems. In [12], Ding et al. investigated the feasibility of the combination of NOMA and FD in a system model, in which uplink and downlink are carried out simultaneously. The provided analytical and simulation results showed that FD-NOMA could offer significant performance gain compared to half-duplex NOMA and orthogonal multiple access (OMA). A novel cooperative FD-NOMA with a two-way relay under both perfect and imperfect SIC was proposed and investigated in [13]. The authors derived the closed-form expressions of outage probability (OP), ergodic capacity (EC), and other parameters under the assumption of imperfect self-interference cancellation. Their results pointed out that OP floors and EC ceilings existed due to the inter-user interference among superposition NOMA symbols and the residual loop-interference caused by imperfect SIC. Being interested in the physical layer security, Cao et al. [14] considered the secure transmission of cooperative FD-NOMA networks under the presence of eavesdroppers. The exact and asymptotic expressions of the secrecy outage probability were derived to measure the secrecy performance. Mohammadi et al. [15] investigated an FD-NOMA with a multi-antenna relay under the cognitive radio environment. They solved the joined power optimization problem of FD relay and access point to maximize the near user’s rate under a constraint that the far user’s rate is above a predetermined threshold. A review of FD-NOMA system models is given in [16], pointing out the opportunities and challenges for application into next-generation radio systems.

In recent years, energy harvesting (EH) technology is a research topic that has attracted much interest from researchers [17,18,19]. EH technology allows network nodes to harvest energy from the surrounding radio frequency (RF) in the radio bandwidth at the receiver to convert it into DC power for next operations [20]. Using the EH technique allows network nodes to extend their operating time, even when wireline power supplies do not power them. This approach is also the research trend towards green communications that many research groups are developing [21]. Interestingly, combining FD-NOMA and EH technique has been carried out in several works such as [22,23,24,25,26] to enhance both energy and spectral efficiency, thus, overcoming the energy and spectral scarcity in the wireless communications systems. Specifically, Cao et al. [22] introduced a novel communication scheme that combines beamforming and EH into a cooperative FD-NOMA system. Three different cases of the self-interference signal in FD operation were investigated. The authors proved that introducing EH not only motivates users to collaborate but also reduces the self-interference impact. Wang et al. [23] proposed a cooperative FD-NOMA system, where the nearby user can be used as a relay with a self-energy recycling protocol, i.e., the relay harvests energy from dedicated energy signal transmitted by a base station while it reuses energy from loop self-interference signal generated in FD operation. The exact and asymptotic expressions for the outage probabilities of users were derived. Numerical results showed that their proposed self-energy recycling FD-NOMA system outperform orthogonal multiple access (OMA)-based system. Yuan et al. [24] also employed near the user as a full-duplex relay and considered the usage EH technique to harvest energy from the RF signal transmitted by the source. With the assumption of imperfect channel state information (CSI) and imperfect SI, the authors solved the energy efficiency maximization problem while guaranteeing the far user’s minimum target rate. Generally, most works use FD relay with a receiving and transmitting antenna due to IoT devices’ limited sizes. To see the effect of the number of antennas on the system quality, Liu et al. [25] considered the FD-NOMA relay system where the near user with one receiving antenna and multiple transmitting antennas acted as an FD relay under full and partial CSI conditions. Considering the cooperative NOMA system using a dedicated FD relay with multiple antennas, Huang et al. [26] formulated and solved the problem of maximizing energy efficiency with the guarantee of quality-of-service (QoS) requirements for two users. Both [25] and [26] solved the energy efficiency problem under the constraints of the performance systems but mainly built optimization problems under the constraints of several system parameters without giving mathematical analysis. Therefore, the influences of several system parameters such as the SI cancellation coefficient and the strength of inter-user interference on the outage probability and ergodic capacity of users was not clearly presented. Guo et al. [27] investigated a NOMA relay system, where the energy-limited FD near user harvest energy from the source then acts as a relay to forward the decoded signal to the far user. Dang et al. [28] proposed three FD cooperative relaying NOMA scheme for device-to-device (D2D) communications and evaluate the proposed schemes through the closed-form outage probability and throughput expressions. However, the authors did not consider interference from the transmitted relay to the near user.

Motivated by the above issues, in this paper, we mathematically analyze an FD-NOMA relay system where the far user is supported by an EH relay to convey its intended signals. The contributions of this paper can be summarized as follows:

- We analyze the performance of a NOMA system where an FD relay assists the communication between the base station and the far user while the near user can communicate with the base station directly. The relay uses the EH technique to harvest the energy of the base station’s signals by using a power splitting protocol. For the practical purpose, the interference from the relay to the near user is taken into consideration.
- We derive the exact analytical expressions of the outage probabilities and ergodic capacities at two destination users in the system under Rayleigh fading channels. We also conduct Monte–Carlo simulations to verify the correctness of the derived mathematical expressions.
- We provide more insights into the effect of the strength of the inter-user interference parameter at the relay and the self-interference cancellation coefficient on the outage probabilities and ergodic capacities at users. Moreover, the optimal value of the power division ratio can be determined by using our theoretical results to achieve the best performance of the considered EH-FD-NOMA relay system.

The rest of the paper is organized as follows. Section 2 describes the considered system and channel models. Section 3 focuses on deriving the exact analytical expressions of the outage probabilities and ergodic capacities of two users. Numerical results and the corresponding discussions are presented in Section 4. Finally, some conclusions are given in Section 5.

For the sake of clarity, we provide in Table 1 the symbols along with their descriptions used in this paper.

## 2. System Model

**Figure 1:**
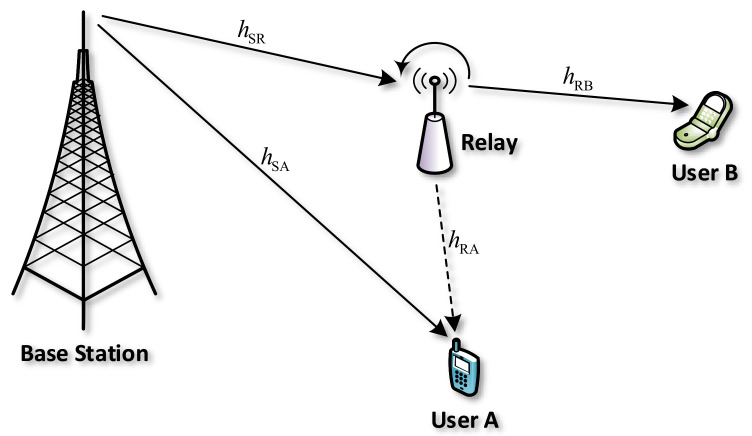
System model of downlink full-duplex (FD) non-orthogonal multiple access (NOMA) relay system with an energy harvesting (EH) relay.

We consider a downlink NOMA relay system, as illustrated in Figure 1. A base station (S) transmits its signals to two users A and B by using the NOMA technique. Since B locates at a long distance, a relay (R) is required to forward signals from S to B. In terms of relaying protocols, R can employ decode-and-forward (DF) or amplify-and-forward (DF). If R uses an AF relaying protocol, it amplifies the noise and errors in the received signals from S and forwards them to A and B. Moreover, R also requires an expensive RF chain to mitigate the coupling effects. If R used DF relaying protocol, it samples the received signals from S and only forwards correctly decoded signals to A and B. For this reason, the DF relaying protocol often provides better system performance than the AF relaying protocol [29,30]. Furthermore, R applies the energy harvesting (EH) technology to collect the energy from the transmitted signal of S and uses that energy for forwarding signal to B. It is worth noticing that R can be equipped with linear/non-linear EH harvester [26,31,32]. In our considered NOMA relay system, to guarantee the quality-of-service (QoS) at the far user, higher transmission power of R is needed. Therefore, R is assumed to employ linear EH harvester so that it can harvest more energy. It is also assumed that S, A, and B in the system are equipped with a single antenna while R is equipped with two antennas for transmitting and receiving in FD mode.

All wireless channels in the system undergo flat, independent, and non-identically distributed Rayleigh fading. Specifically, the channel gain from node X to node Y, ${\left|h_{\mathrm{XY}}\right|}^{2}$, is an exponential distribution variable whose the probability distribution density function (PDF) and the cumulative probability distribution function (CDF) are, respectively, given by

(1) $$\begin{array}{c} f_{{\left|h_{\mathrm{XY}}\right|}^{2}}\left(z\right)\;=\;\frac{1}{\lambda_{\mathrm{XY}}}\exp\left(-\frac{z}{\lambda_{\mathrm{XY}}}\right), \end{array}$$

and

(2) $$\begin{array}{c} F_{{\left|h_{\mathrm{XY}}\right|}^{2}}\left(z\right)\;=\;1-\exp\left(-\frac{z}{\lambda_{\mathrm{XY}}}\right). \end{array}$$

According to the coding principle of NOMA, S applies the superposition coding technique to combine two independent signals and then transmits this combined signal to A and B, i.e.,

(3) $$\begin{array}{c} x_{S}\;\left[n\right]\;=\;\sqrt{P_{S}a_{1}}x_{A}\;\left[n\right]\;+\;\sqrt{P_{S}a_{2}}x_{B}\;\left[n\right], \end{array}$$

where $x_{A}$ and $x_{B}$ are intended signals for A and B, respectively; $a_{1}$ and $a_{2}$ are the power allocation factor for $x_{A}$ and $x_{B}$.

Therefore, the received signal at R is

(4) $$\begin{array}{c} y_{R}\;\left[n\right]\;=\;h_{\mathrm{BR}}x_{S}\;\left[n\right]\;+\;\sqrt{P_{R}}h_{\mathrm{RR}}x_{B}\;\left[n-\tau \right]\;+\;n_{R}\;\left[n\right], \end{array}$$

where $n_{R}\left[n\right]\;∼\;\mathrm{CN}\left(0,\sigma_{R}^{2}\right)$ represents the additive white Gaussian noise (AWGN); $h_{\mathrm{RR}}$ is the loop interference channel at R; τ, τ≥1, is an integer, representing the time delay (in the number of time slots) due to the signal processing in FD mode [33]. In the first τ time slots, R operates in the HD mode because there is no symbol to transmit. Hence, $x_{B}$ and $x_{A}$ are decoded at A by using SIC technique without being interfered by R. However, from the next $\left(\tau +1\right)$ time slot, R operates in the FD mode. Then, A is affected by the interference from R and the signal transmitted from R to B is delayed for τ time slots, i.e., $x_{B}\left[n-\tau \right]$.

The power splitting protocol is applied at R to harvest the power of the received signal $y_{R}\left[n\right]$. Specifically, the total power of the received signal at R is divided into two parts: one for the energy harvesting and another for signal decoding. Let α, $\alpha \in \left(0,1\right)$, be the power division ratio. Then, a part of the received signal at R for EH can be expressed as [26]

(5) $$\begin{array}{c} \;y_{R\to \mathrm{EH}}\;=\;\sqrt{1-\alpha }y_{R}\;\left[n\right]\;=\;\sqrt{1\;-\;\alpha }\;\left(\;h_{\mathrm{BR}}x_{S}\;\left[n\right]\;+\;\sqrt{\;P_{R}}h_{\mathrm{RR}}x_{B}\;\left[n\;-\;\tau \right]\;+\;n_{R}\;\left[n\right]\right). \end{array}$$

Consequently, the harvested energy at R is

(6) $$\begin{array}{c} E_{R}\;=\;\xi \;\left(1-\alpha \right)\;\left(P_{S}{\left|h_{\mathrm{SR}}\right|}^{2}+P_{R}{\left|h_{\mathrm{RR}}\right|}^{2}+\sigma_{R}^{2}\right), \end{array}$$

where ξ is the energy conversion efficiency.

Suppose all the energy that R harvests is used to forward signals $x_{B}$ to the far user B. Then, the transmission power of R is determined as

(7) $$\begin{array}{c} P_{R}\;=\;\frac{\xi \;\left(1-\alpha \right)\left(P_{S}{\left|h_{\mathrm{SR}}\right|}^{2}+P_{R}{\left|h_{\mathrm{RR}}\right|}^{2}\;+\;\sigma_{R}^{2}\right)T}{T-\tau }, \end{array}$$

where T denotes the signal transmission cycle.

On the other hand, a part of the received signal at R for information decoding (ID) is given by

(8) $$\begin{array}{ll} y_{R\to \mathrm{ID}} & =\;\sqrt{\alpha }y_{R}\;\left[n\right]\\ & =\sqrt{\alpha }\;\left(h_{\mathrm{BR}}x_{S}\;\left[n\right]\;+\;\sqrt{P_{R}}h_{\mathrm{RR}}x_{B}\;\left[n-\tau \right]\;+\;n_{R}\;\left[n\right]\right). \end{array}$$

According to the decoding principle of NOMA, R decodes $x_{B}$ by treating $x_{A}$ as interference. Then, the signal-to-interference-plus-noise ratio (SINR) to decode $x_{B}$ at R is determined as [26]

(9) $$\begin{array}{c} \gamma_{R,x_{B}}=\frac{\alpha P_{S}a_{2}{\left|h_{\mathrm{SR}}\right|}^{2}}{\alpha \left(P_{S}a_{1}{\left|h_{\mathrm{SR}}\right|}^{2}\;+\;P_{R}{\left|h_{\mathrm{RR}}\right|}^{2}+\sigma_{R}^{2}\right)\;+\;\delta_{R}^{2}}, \end{array}$$

where $\delta_{R}^{2}$ is the variance of the AWGN noise generated during signal decoding.

Assuming that R is able to recognize $x_{B}\left[n-\tau \right]$ from the previous decoding process, thus R can use SI cancellation technique to eliminate $x_{B}\left[n-\tau \right]$ in the loop interference. However, it is difficult to eliminate $x_{B}\left[n-\tau \right]$ completely, so there is usually a residual self-interference (RSI). This RSI is modeled as a random variable having a complex Gaussian distribution with zero mean and variance $I_{R}$ [34,35].

The SINR for decoding $x_{B}$ at R is given by

(10) $$\begin{array}{c} \gamma_{R,x_{B}}\;=\;\frac{P_{S}a_{2}{\left|h_{\mathrm{SR}}\right|}^{2}}{\left(P_{S}a_{1}{\left|h_{\mathrm{SR}}\right|}^{2}\;+\;I_{R}\;+\;\sigma_{R}^{2}\right)\;+\;\frac{\delta_{R}^{2}}{\alpha }}, \end{array}$$

and the received signal at B can be expressed as

(11) $$\begin{array}{c} y_{B}=\sqrt{P_{R}}h_{\mathrm{RB}}x_{B}\;\left[n-\tau \right]\;+\;n_{B}\;\left[n\right], \end{array}$$

where $n_{B}\;\left[n\right]\;∼\;\mathrm{CN}\;\left(0,\sigma_{B}^{2}\right)$ is the AWGN at B.

Meanwhile, the SINR for decoding $x_{B}$ at B is given by

(12) $$\begin{array}{c} \gamma_{x_{B}}\;=\;\frac{P_{R}{\left|h_{\mathrm{RB}}\right|}^{2}}{\sigma_{B}^{2}}, \end{array}$$

and the received signal at A is presented as

(13) $$\begin{array}{c} y_{A}\;\left[n\right]\;=\;h_{\mathrm{SA}}x_{S}\;\left[n\right]\;+\;\sqrt{P_{R}}h_{\mathrm{RA}}x_{B}\;\left[n\;-\;\tau \right]\;+\;n_{A}\;\left[n\right], \end{array}$$

where $n_{A}\;\left[n\right]\;∼\;\mathrm{CN}\;\left(0,\sigma_{A}^{2}\right)$ is the AWGN at A.

According to the decoding principle of NOMA, A first decodes $x_{B}$ by treating $x_{A}$ as interference. The SINR to decode $x_{B}$ at A is given by

(14) $$\begin{array}{c} \gamma_{x_{B}\to x_{A}}\;=\;\frac{P_{S}a_{2}{\left|h_{\mathrm{SA}}\right|}^{2}}{P_{S}a_{1}{\left|h_{\mathrm{SA}}\right|}^{2}\;+\;P_{R}{\left|h_{\mathrm{RA}}\right|}^{2}\;+\;\sigma_{A}^{2}}. \end{array}$$

Note that A already knows $x_{B}\;\left[n-\tau \right]$ due to the previous decoding process, so it can use the self-interference cancellation technique to eliminate $x_{B}\;\left[n-\tau \right]$ as [36]. However, it is difficult to wholly eliminate the signal $x_{B}\left[n-\tau \right]$. Therefore, the $h_{RA}$ channel is modeled as the inter-user interference channel with parameter *k* represents the strength of inter-user interference, i.e., $h_{\mathrm{RA}}\;∼\;\mathrm{CN}\;\left(0,\;k\lambda_{\mathrm{RA}}\right)$ [15].

After successfully decoding $x_{B}$, A removes $x_{B}$ and performs decoding $x_{A}$ in the second step with the SINR is determined as

(15) $$\begin{array}{c} \gamma_{x_{A}}=\frac{P_{S}a_{1}{\left|h_{\mathrm{SA}}\right|}^{2}}{P_{R}{\left|h_{\mathrm{RA}}\right|}^{2}\;+\;\sigma_{A}^{2}}. \end{array}$$

## 3. Performance Analysis

In this section, we analyze the performance of the considered system with two important metrics: the outage probability and ergodic capacity at two terminals A and B.

### 3.1. Outage Probability

#### 3.1.1. The Outage Probability at A, ${\mathrm{OP}}_{x_{A}}$

Near user A is not in outage when it can decode both signal $x_{A}$ and $x_{B}$ received from S. Consequently, the OP at A, denoted by ${\mathrm{OP}}_{x_{A}}$, is expressed as

(16) $$\begin{array}{c} {\mathrm{OP}}_{x_{A}}\;=\;1-\mathrm{Pr}\left(\gamma_{x_{B}\to x_{A}}>\gamma_{B},\gamma_{x_{A}}>\gamma_{A}\right), \end{array}$$

where $\gamma_{B}\;=\;2^{R_{B}}-1$, $\gamma_{A}\;=\;2^{R_{A}}-1$; $R_{A}$ and $R_{B}$ are the desired data rate of $x_{A}$ and $x_{B}$ at A and B, respectively.

The OP of $x_{A}$ at near user A is determined in the following Theorem 1.

**Theorem** **1.** *The exact analytical expression for the OP of near user A in the considered FD-NOMA with EH relay is given by*

(17) $$\begin{array}{c} OP_{x_{A}}\;=\;1-\exp\left(-\frac{\sigma_{A}^{2}}{\varphi }\right)\;+\;\exp\left(\frac{c}{b\lambda_{\mathrm{SR}}}-\frac{\sigma_{A}^{2}}{\varphi }\;+\;\frac{\lambda_{\mathrm{SA}}}{2b\varphi \lambda_{\mathrm{SR}}k\lambda_{\mathrm{RA}}}\right)\;\times \;W_{-1,\frac{1}{2}}\left(\frac{\lambda_{\mathrm{SA}}}{b\varphi \lambda_{\mathrm{SR}}k\lambda_{\mathrm{RA}}}\right), \end{array}$$

*where $W_{\alpha ,\beta }\left(\cdot \right)$ is the Whittaker function [37] Equation (9.220),*

(18) $$\varphi =\left\{\begin{array}{l} \frac{P_{S}\left(a_{2}-a_{1}\gamma_{B}\right)}{\gamma_{B}},\;\mathrm{if}\;\gamma_{B}<\frac{a_{2}}{a_{1}}<\gamma_{B}\;+\;\frac{\gamma_{B}}{\gamma_{A}}\\ \frac{P_{S}a_{1}}{\gamma_{A}},\;\mathrm{if}\;\frac{a_{2}}{a_{1}}>\gamma_{B}\;+\;\frac{\gamma_{B}}{\gamma_{A}} \end{array}\right.,$$

*and ${\mathrm{OP}}_{x_{A}}=1$ if $a_{2}/a_{1}\leq \gamma_{B}$.*

**Proof.**  In the case of $a_{2}/a_{1}\leq \gamma_{B}$, we can easily prove that ${\mathrm{OP}}_{x_{A}}=1$. In the case of $a_{2}/a_{1}>\gamma_{B}$, from (16), the OP of $x_{A}$ at A can be computed as

(19) $$\begin{array}{cc} {\mathrm{OP}}_{x_{A}} & =1-\mathrm{Pr}\left(\frac{P_{S}a_{2}{\left|h_{\mathrm{SA}}\right|}^{2}}{P_{S}a_{1}{\left|h_{\mathrm{SA}}\right|}^{2}+P_{R}{\left|h_{\mathrm{RA}}\right|}^{2}+\sigma_{A}^{2}}>\gamma_{B},\frac{P_{S}a_{1}{\left|h_{\mathrm{SA}}\right|}^{2}}{P_{R}{\left|h_{\mathrm{RA}}\right|}^{2}+\sigma_{A}^{2}}>\gamma_{A}\right)\\ \; & =1-\int_{0}^{\infty }\mathrm{Pr}\left(P_{R}{\left|h_{\mathrm{RA}}\right|}^{2}<\Psi \right)f_{{\left|h_{\mathrm{SA}}\right|}^{2}}\left(z\right)dz, \end{array}$$

where

(20) $$\begin{array}{c} \;\Psi \;=\;\min\;\left(\;\frac{P_{S}\;\left(a_{2}\;-\;a_{1}\;\gamma_{B}\right)}{\gamma_{B}}{\left|h_{\mathrm{SA}}\;\right|}^{2}\;-\;\sigma_{A}^{2},\frac{P_{S}a_{1}}{\gamma_{A}}{\left|h_{\mathrm{SA}}\right|}^{2}\;-\;\sigma_{A}^{2}\;\right)\;.\; \end{array}$$

Therefore, we have

(21) $$\begin{array}{c} {\mathrm{OP}}_{x_{A}}=\;\left\{\begin{array}{c} 1\;-\;\int_{0}^{\infty }\;\mathrm{Pr}\;\left(P_{R}{\left|h_{\mathrm{RA}}\right|}^{2}\;<\;\frac{P_{S}\left(a_{2}\;-\;a_{1}\gamma_{B}\right)}{\gamma_{B}}z\;-\;\sigma_{A}^{2}\right)f_{{\left|h_{\mathrm{SA}}\right|}^{2}}\;\left(z\right)\;dz,\;\mathrm{if}\;\gamma_{B}\;<\;\frac{a_{2}}{a_{1}}\;<\;\gamma_{B}+\frac{\gamma_{B}}{\gamma_{A}}\\ 1\;-\;\int_{0}^{\infty }\;\mathrm{Pr}\left(P_{R}{\left|h_{\mathrm{RA}}\right|}^{2}\;<\;\frac{P_{S}a_{1}}{\gamma_{A}}z\;-\;\sigma_{A}^{2}\right)f_{{\left|h_{\mathrm{SA}}\right|}^{2}}\;\left(z\right)\;dz,\;\mathrm{if}\;\frac{a_{2}}{a_{1}}\;>\;\gamma_{B}\;+\;\frac{\gamma_{B}}{\gamma_{A}} \end{array}\right. \end{array}$$

For the sake of convenience, we perform the following integration

(22) $$\begin{array}{c} I_{1}=\int_{0}^{\infty }\mathrm{Pr}\left(P_{R}{\left|h_{\mathrm{RA}}\right|}^{2}<\varphi z-\sigma_{A}^{2}\right)f_{{\left|h_{\mathrm{SA}}\right|}^{2}}\left(z\right)dz. \end{array}$$

On the other hand, the transmission power of R can be rewritten as

(23) $$\begin{array}{c} P_{R}=b{\left|h_{\mathrm{SR}}\right|}^{2}+c, \end{array}$$

where $b=\frac{\xi \left(1-\alpha \right)T}{T-\tau }P_{S}$, $c=\frac{\xi \left(1-\alpha \right)\left(I_{R}+\sigma_{R}^{2}\right)T}{T-\tau }$.

Using the results of Appendix A, combining (21), (22) and (A3), we have the exact expression of ${\mathrm{OP}}_{x_{A}}$ as (17). □

#### 3.1.2. The Outage Probability at B, ${\mathrm{OP}}_{x_{B}}$

Far user B is in outage when either R cannot decode $x_{B}$ received from S or B cannot decode $x_{B}$ forwarded by R to B. Therefore, the OP at B (denoted by ${\mathrm{OP}}_{x_{B}}$) can be expressed as

(24) $$\begin{array}{c} {\mathrm{OP}}_{x_{B}}\;=\;1-\mathrm{Pr}\;\left(\gamma_{R,x_{B}}\;>\;\gamma_{B},\gamma_{x_{B}}\;>\;\gamma_{B}\right)\;=\;1-\mathrm{Pr}\;\left(\;\frac{P_{S}a_{2}{\left|h_{\mathrm{SR}}\right|}^{2}}{P_{S}a_{1}{\left|h_{\mathrm{SR}}\right|}^{2}\;+\;m}\;>\;\gamma_{B},\frac{\left(b{\left|h_{\mathrm{SR}}\right|}^{2}\;+\;c\right){\left|h_{\mathrm{RB}}\right|}^{2}}{\sigma_{B}^{2}}\;>\;\gamma_{B}\right), \end{array}$$

where $m=I_{R}+\sigma_{R}^{2}+\delta_{R}^{2}/\alpha$.

The OP of $x_{B}$ at far user B is determined in the following Theorem 2.

**Theorem** **2.** *The exact analytical expressions for the OP of far user B in the considered FD-NOMA with EH relay is given by*

(25) $$\begin{array}{cc} OP_{x_{B}} & =1-\exp\left(-\frac{x_{0}}{\lambda_{\mathrm{RB}}}-\frac{m\gamma_{B}}{P_{S}\left(a_{2}-a_{1}\gamma_{B}\right)\lambda_{\mathrm{SR}}}\right)-\frac{1}{\lambda_{\mathrm{SR}}}\exp\left(-\frac{c}{b\lambda_{\mathrm{SR}}}\right)\sum_{n=0}^{N}\frac{{\left(-1\right)}^{n}}{n!}{\left(\frac{1}{\lambda_{\mathrm{RB}}}\right)}^{n}{\left(\frac{\gamma_{B}\sigma_{B}^{2}}{b\lambda_{\mathrm{SR}}}\right)}^{n/2}\\ & \;\times \;{\left(x_{0}\right)}^{1+n/2}\exp\;\left(\;-\frac{\gamma_{B}\sigma_{B}^{2}}{2b\lambda_{\mathrm{SR}}x_{0}}\;\right)\;W_{-1-\frac{n}{2},\frac{n+1}{2}}\;\left(\frac{\gamma_{B}\sigma_{B}^{2}}{b\lambda_{\mathrm{SR}}x_{0}}\right)\;,\; \end{array}$$

*where N is the number of truncated terms in the series expansion, and*

(26) $$\begin{array}{c} x_{0}=\frac{P_{S}\left(a_{2}-a_{1}\gamma_{B}\right)\gamma_{B}\sigma_{B}^{2}}{mb\gamma_{B}+cP_{S}\left(a_{2}-a_{1}\gamma_{B}\right)}. \end{array}$$

**Proof.**  See Appendix B. □

### 3.2. Ergodic Capacity

#### 3.2.1. Ergodic Capacity of Signal $x_{A}$ at A, $C_{x_{A}}$

The EC of $x_{A}$ on the link from S to A is given by

(27) $$\begin{array}{c} C_{x_{A}}=\int_{0}^{\infty }\log_{2}\left(1+x\right)f_{\gamma_{x_{A}}}\left(x\right)\;dx, \end{array}$$

where $f_{\gamma_{x_{A}}}$ denotes the PDF of $\gamma_{x_{A}}$.

Using the integration by part, we can express (27) in terms of the CDF of $\gamma_{x_{A}}$ as

(28) $$\begin{array}{c} C_{x_{A}}=\frac{1}{\ln2}\int_{0}^{\infty }\frac{1-F_{\gamma_{x_{A}}}\left(x\right)}{1+x}dx, \end{array}$$

where $F_{\gamma_{x_{A}}}$ is the CDF of $\gamma_{x_{A}}$.

To find the expression of the EC of $x_{A}$, we first derive $F_{\gamma_{x_{A}}}$, then calculate the integral in (28). The EC of $x_{A}$ is determined in the following Theorem 3.

**Theorem** **3.** *The exact analytical expression of the EC of $x_{A}$ at A in the considered FD-NOMA with EH relay is given by*

(29) $$\begin{array}{cc} C_{x_{A}} & =\;\frac{1}{\ln2}\;\left[\exp\;\left(-\vartheta \right)\;\mathrm{Ei}\;\left(\vartheta \right)-\exp\;\left(\vartheta \right)\;\mathrm{Ei}\;\left(-\vartheta \right)\right]\;+\;\frac{1}{\ln2}\frac{1}{\zeta /\vartheta +1}\;\left[\exp\;\left(\zeta \right)\;\mathrm{Ei}\;\left(-\zeta \right)-\exp\;\left(-\zeta \right)\;\mathrm{Ei}\;\left(\zeta \right)\right]\\ \; & +\;\frac{\Psi P_{S}a_{1}}{2c}\;\left[A\exp\;\left(\vartheta \right)\;\mathrm{Ei}\;\left(-\vartheta \right)\;+\;B\exp\;\left(\theta \vartheta \right)\;\mathrm{Ei}\;\left(-\theta \vartheta \right)\right]\;+\;\frac{\Psi P_{S}a_{1}}{2c}\;\left(1-C\right)\lambda_{\mathrm{SA}}^{2}\;\left[\vartheta \exp\;\left(\theta \vartheta \right)\;\mathrm{Ei}\;\left(-\theta \vartheta \right)\;+\;\frac{1}{\theta }\right], \end{array}$$

*where $\vartheta =\frac{\sigma_{A}^{2}}{P_{S}a_{1}\lambda_{\mathrm{SA}}}$, $\zeta =\frac{\sigma_{A}^{2}}{ck\lambda_{\mathrm{RA}}}$, $\theta =\frac{P_{S}a_{1}\lambda_{\mathrm{SA}}\left(y_{i}+1\right)}{2ck\lambda_{\mathrm{RA}}}$, $A=\frac{1}{{\left(\theta -1\right)}^{2}}$, $B=-\frac{1}{{\left(\theta -1\right)}^{2}}$, $C=\frac{1-\theta }{{\left(\theta -1\right)}^{2}}$, and $Ei\left(\cdot \right)$ denotes the exponential integral function [37] Equation (8.211).*

**Proof.**  See Appendix C. □

#### 3.2.2. Ergodic Capacity of Signal $x_{B}$ at B, $C_{x_{B}}$

Setting $X=\min\left(\gamma_{x_{B}}^{R},\gamma_{x_{B}}^{B}\right)$. Then, the CDF of *X* (denoted by $F_{X}\left(x\right)$) is expressed as

(30) $$\begin{array}{c} F_{X}\left(x\right)\;=\;\mathrm{Pr}\;\left(\;\min\;\left(\gamma_{x_{B}}^{R},\gamma_{x_{B}}^{B}\right)\;<\;x\right). \end{array}$$

Hence, the EC of signal $x_{B}$ can be computed as

(31) $$\begin{array}{c} C_{x_{B}}=\int_{0}^{\infty }\log_{2}\;\left(1+x\right)\;f_{X}\;\left(x\right)\;dx, \end{array}$$

where $f_{X}\;\left(x\right)$ is the PDF of *X*.

After using the integration by part, the EC of $x_{B}$ at B can be expressed as

(32) $$\begin{array}{c} C_{x_{B}}\;=\;\frac{1}{\ln2}\int_{0}^{\infty }\frac{1-F_{X}\;\left(x\right)}{1+x}dx. \end{array}$$

To obtain $C_{x_{B}}$, similar to in [38], we use the Gaussian–Chebyshev quadrature approach as an effective approximation method to calculate the integration of a function f(x) over an interval (a,b), i.e.,

(33) $$\begin{array}{c} \int_{a}^{b}f\;\left(x\right)\;dx\;\approx \;\frac{b-a}{2}\sum_{i=1}^{n}\omega_{n}\sqrt{1-y_{i}^{2}}f\;\left(x_{i}\right), \end{array}$$

where $x_{i}=\frac{b-a}{2}y_{i}+\frac{b+a}{2}$, $y_{i}=\cos\left(\frac{2i-1}{2n}\pi \right)$ and $\omega_{n}=\frac{\pi }{n}$. Then, $C_{x_{B}}$ is determined in the following Theorem 4.

**Theorem** **4.** *The approximate analytical expression for the EC of $x_{B}$ at B in the considered FD-NOMA with EH relay is given by*

(34) $$\begin{array}{cc} C_{x_{B}} & =\frac{u}{2\ln2}\sum_{k=1}^{K}\omega_{K}\sqrt{1-y_{k}^{2}}\frac{1}{1+x_{k}}\exp\;\left(-\eta -\frac{mx_{k}}{P_{S}\;\left(a_{2}-a_{1}x_{k}\right)\;\lambda_{\mathrm{SR}}}\right)\\ \; & +\;\frac{u}{4\ln2}\sum_{i=1}^{N}\sum_{k=1}^{K}\frac{\omega_{N}\omega_{K}\eta }{\lambda_{\mathrm{RB}}\;\left(1+x_{k}\right)}\sqrt{1-y_{i}^{2}}\sqrt{1-y_{k}^{2}}\exp\;\left(\frac{c}{b\lambda_{\mathrm{SR}}}\right)\;\exp\;\left(-2\varpi +\frac{1}{2}\eta \;\left(y_{i}+1\right)\right), \end{array}$$

*where K and N are the complexity-accuracy trade-off parameters, and*

(35) $$\begin{array}{cc} \eta & =\frac{P_{S}\sigma_{B}^{2}\;\left(a_{2}-a_{1}x_{k}\right)\;x_{k}}{mbx_{k}+cP_{S}\;\left(a_{2}-a_{1}x_{k}\right)}, \end{array}$$

(36) $$\begin{array}{cc} \varpi & \;=\;\frac{\left(mbx_{k}+cP_{S}\;\left(a_{2}-a_{1}x_{k}\right)\right)}{b\lambda_{\mathrm{SR}}P_{S}\;\left(y_{i}+1\right)\;\left(a_{2}-a_{1}x_{k}\right)}. \end{array}$$

**Proof.**  See Appendix D. □

## 4. Numerical Results

In this section, we provide analysis results together with Monte–Carlo simulation results to verify the derived mathematical expressions. It is assumed that all nodes in the considered system locate on a 2D plane. Specifically, the locations of all nodes are S(0, 0), R(0.8, 0), A (−0.3, 0.7), B(1.5, 0). We can see that since R locates approximately in the middle between S and B, the best outage performance can be achieved, as demonstrated in [39,40]. It is noticed that the communication between the transmitter and the receiver only has one link. Thus, the transmitter does not require instantaneous CSI. Instead, S in our considered FD-NOMA relay system needs to know the average $|h_{\mathrm{SR}}{|}^{2}$ and $|h_{\mathrm{SA}}{|}^{2}$ to allocate power for $x_{A}$ and $x_{B}$. These average values depend on the distance between S and R and the distance between S and A, respectively. On the other hand, since the locations of all nodes in our considered system are fixed, the power allocation coefficients for $x_{A}$ and $x_{B}$ are also fixed. Consequently, considering the imperfect CSI may not necessary. Letting $d_{XY}$ be the distance between X and Y, we have $\lambda_{XY}={d_{XY}}^{-\beta }$ for free-space path-loss transmission, where β is the path-loss exponent, 2≤β≤6. In all evaluating scenarios, the system parameters are set as follows: β=3, $\gamma_{A}=1.5$, $\gamma_{B}=2$, α=0.6, ξ=0.8, $\sigma_{A}^{2}=\sigma_{B}^{2}=\sigma_{R}^{2}=\delta_{R}^{2}=\sigma^{2}$, and k=0.03. The average SNR is defined as $P_{S}/\sigma^{2}$.

**Figure 2:**
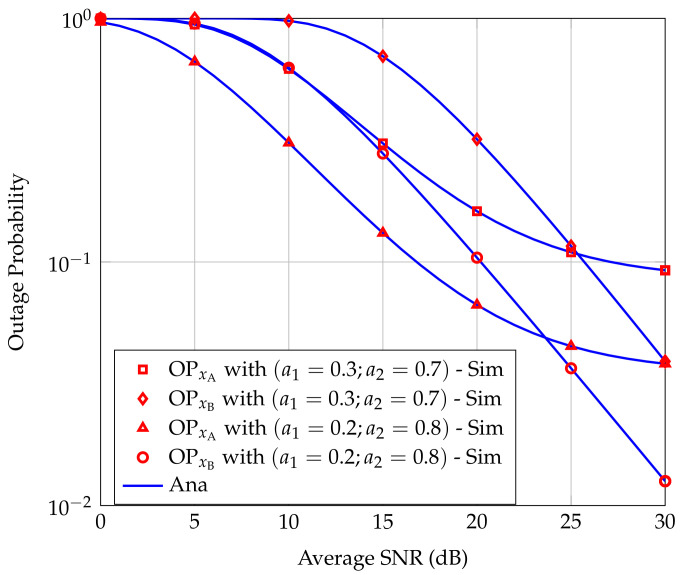
Outage probabilities at users A and B versus average SNR for different power allocation strategies.

Figure 2 presents ${\mathrm{OP}}_{x_{A}}$ and ${\mathrm{OP}}_{x_{B}}$ as functions of the average SNR for two power allocation strategies, i.e., $\left(a_{1}=0.3,a_{2}=0.7\right)$ and $\left(a_{1}=0.2,a_{2}=0.8\right)$. From Figure 2, we can see that the simulation results are in good agreement with the analysis results, confirming the correctness of the derivation approach. Moreover, as the ratio $a_{2}/a_{1}$ is larger, the outage performance of the considered FD-NOMA relay system is better. However, the ratio $a_{2}/a_{1}$ cannot be arbitrarily increased because when $a_{1}$ is too small, the ability to successfully decode the received signals at A will reduce. With our analytical results, this technical challenge can be solved by using numerical methods to find the optimal value of ratio $a_{2}/a_{1}$ for corresponding system parameters. Furthermore, ${\mathrm{OP}}_{x_{A}}$ is remarkably lower than ${\mathrm{OP}}_{x_{B}}$ in the low SNR regime. In contrast, in the high SNR regime, ${\mathrm{OP}}_{x_{B}}$ decreases rapidly while ${\mathrm{OP}}_{x_{A}}$ decreases slowly and reach a floor value.

**Figure 3:**
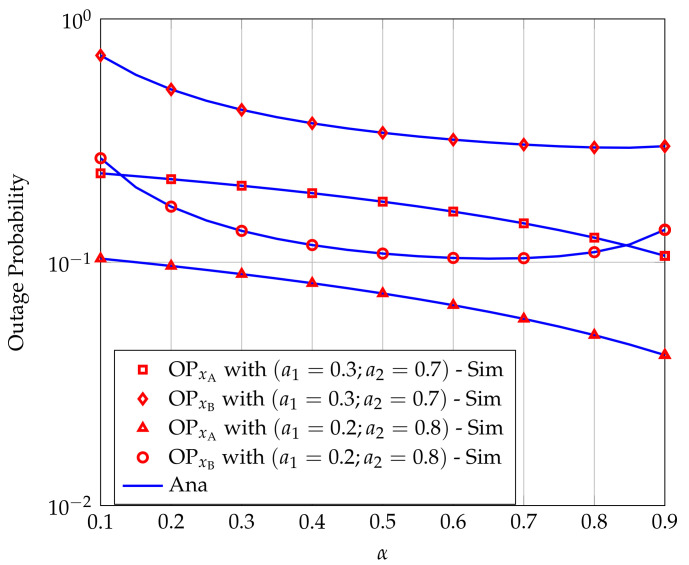
Effect of α on the outage probabilities (OP) at users A and B for different power allocation strategies.

Figure 3 shows the effect of the power division ratio α on ${\mathrm{OP}}_{x_{A}}$ and ${\mathrm{OP}}_{x_{B}}$ for different power allocation strategies. We can see that α significantly affects both ${\mathrm{OP}}_{x_{A}}$ and ${\mathrm{OP}}_{x_{B}}$. Moreover, larger α results in smaller ${\mathrm{OP}}_{x_{A}}$. It is because increasing α makes the energy harvested at R decrease. Consequently, the interference power at A cause by the transmitter at R decreases, then ${\mathrm{OP}}_{x_{A}}$ decreases. However, when increasing α, ${\mathrm{OP}}_{x_{B}}$ does not decrease as monotonically as ${\mathrm{OP}}_{x_{A}}$ but tends to increase when α is larger than a specified value. It is because increasing α the energy harvested at R decreases, leading to the transmission power of R reduces. Therefore, the SNR of the received signal at B is lower, i.e., ${\mathrm{OP}}_{x_{B}}$ is higher.

**Figure 4:**
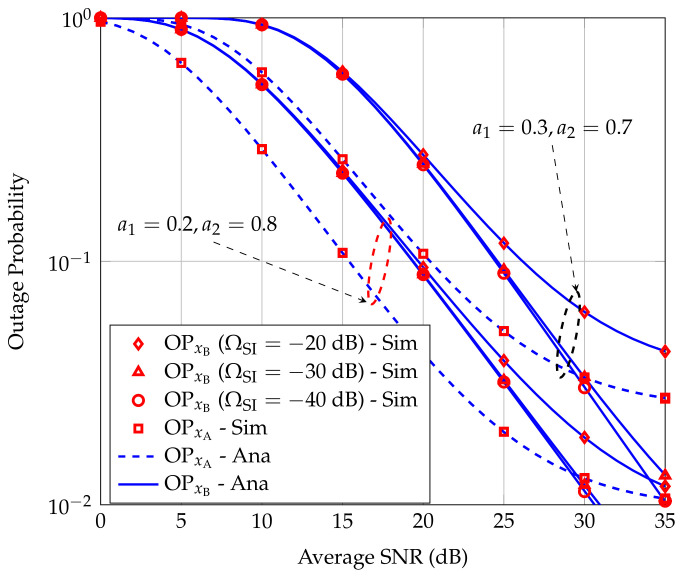
Effect of self-interference (SI) cancellation capacity of R on the OPs at users A and B.

To study the effect of SI suppression technique at R on ${\mathrm{OP}}_{x_{A}}$ and ${\mathrm{OP}}_{x_{B}}$, we define a SI cancellation coefficient, denoted by $\Omega_{\mathrm{SI}}$, to indicate the SI cancellation capacity, i.e., $I_{R}=\Omega_{\mathrm{SI}}P_{R}$. Figure 4 presents ${\mathrm{OP}}_{x_{A}}$ and ${\mathrm{OP}}_{x_{B}}$ when the $\Omega_{\mathrm{SI}}$ varies as −20 dB, −30 dB, and −40 dB in two typical power allocation strategies: $\left(a_{1}=0.2,a_{2}=0.8\right)$ and $\left(a_{1}=0.3,a_{2}=0.7\right)$. We can see that $\Omega_{\mathrm{SI}}$ does not affect ${\mathrm{OP}}_{x_{A}}$ but greatly influences ${\mathrm{OP}}_{x_{B}}$. When $\Omega_{\mathrm{SI}}=-40$ dB, ${\mathrm{OP}}_{x_{B}}$ is close to the floor value. In other words, when $\Omega_{\mathrm{SI}}$ increases, e.g., $\Omega_{\mathrm{SI}}=-50$ dB, ${\mathrm{OP}}_{x_{B}}$ is almost unchanged in comparison with that when $\Omega_{\mathrm{SI}}=-40$ dB. Thus, in this case, we should choose $\Omega_{\mathrm{SI}}=-40$ dB to obtain ${\mathrm{OP}}_{x_{B}}$ close to the best value. Such $\Omega_{\mathrm{SI}}$ can be achieved in practice because the authors in [41] reported that $\Omega_{\mathrm{SI}}$ in FD operation could theoretically reach −110 dB.

**Figure 5:**
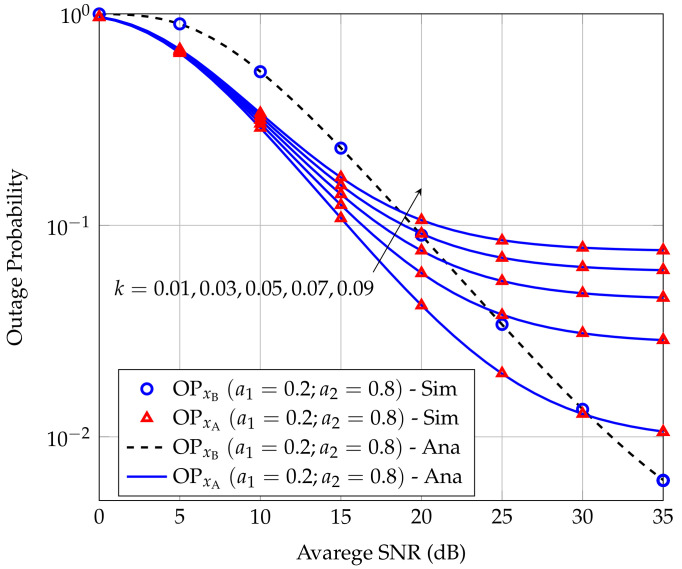
Effect of *k* on the OPs at users A and B.

Figure 5 depicts the effect of inter-user interference on the ${\mathrm{OP}}_{x_{A}}$ and ${\mathrm{OP}}_{x_{A}}$ when its strength *k* varies from 0.01 to 0.09. We can see that *k* does not effect ${\mathrm{OP}}_{x_{B}}$ but greatly affects ${\mathrm{OP}}_{x_{A}}$. Due to the fact that smaller *k* means smaller interference power at A caused by R. Thus, the ${\mathrm{OP}}_{x_{A}}$ is better. Furthermore, when *k* is very small, the interference caused by R at A is negligible, then A almost achieves full diversity.

**Figure 6:**
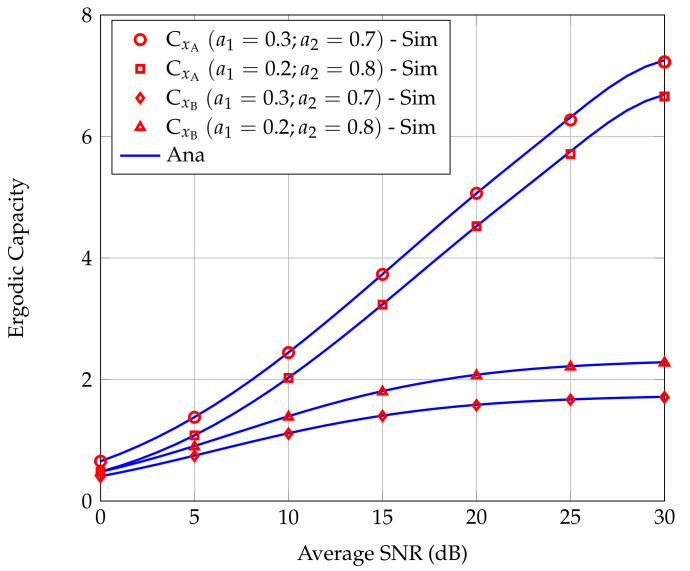
ECs of users A and B versus average SNR for different power allocation strategies.

Figure 6 show $C_{x_{A}}$ and $C_{x_{B}}$ as the functions of the average SNR in two power allocation strategies, i.e., $\left(a_{1}=0.2,a_{2}=0.8\right)$ and $\left(a_{1}=0.3,a_{2}=0.7\right)$. We can see that the simulation results confirm the correctness of the analytical analysis results. The accuracy of analytical results depends on the value of *N* and *K*. To obtain Figure 6, we set N=K=10. From Figure 6, we can see that when increasing $a_{2}/a_{1}$, $C_{x_{B}}$ increases while $C_{x_{A}}$ decreases. Thus, depending on the service requirements at B, an appropriate ratio $a_{2}/a_{1}$ should be chosen to satisfy the conditions $a_{2}-a_{1}\gamma_{B}>0$ and ensure the service quality requirements at A. Generally, $C_{x_{A}}$ is larger than $C_{x_{B}}$ and increases rapidly with the average signal transmission power.

**Figure 7:**
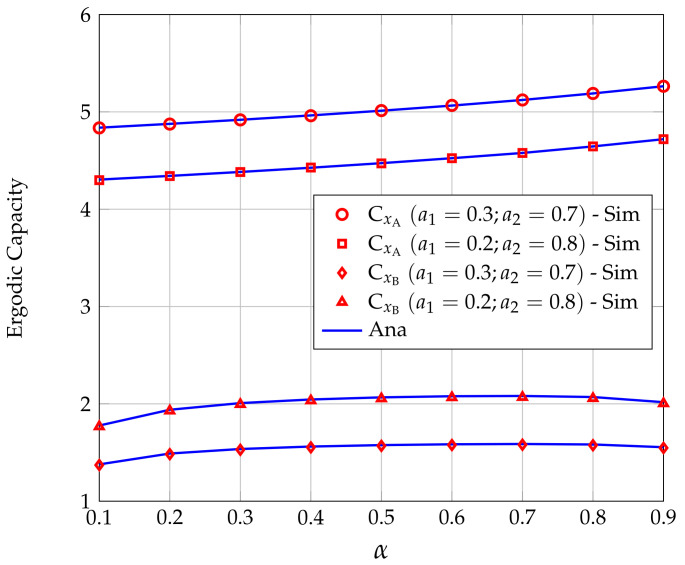
Ergodic capacities (EC) of users A and B versus α for different power allocation strategies.

Figure 7 presents the effect of the power division ratio α on $C_{x_{A}}$ and $C_{x_{B}}$ for two power allocation scenarios, i.e., $\left(a_{1}=0.2,a_{2}=0.8\right)$ and $\left(a_{1}=0.3,a_{2}=0.7\right)$. We see that $C_{x_{A}}$ almost linearly increases with α. The reason behind this feature is that, as α increases, the energy harvested at R decreases, then the transmission power of R is lower, resulting in lower interference caused by R to A. In contrast, $C_{x_{B}}$ does not increase linearly with α but reaches its maximum value when α is in the range of 0.5 to 0.7. The value of α at which $C_{x_{B}}$ reaches the maximum value can be found numerically based on the analysis results given in (34).

**Figure 8:**
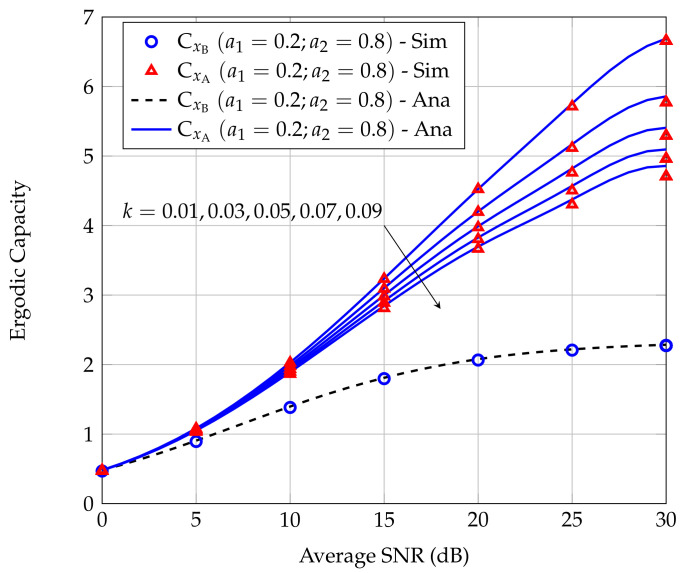
Effect of the strength *k* of inter-user interference on the ECs of users A and B.

Figure 8 shows the effect of the strength *k* of inter-user interference on $C_{x_{A}}$ and $C_{x_{B}}$. The value of *k* increases from 0.1 to 0.9. We can see that as *k* increases, $C_{x_{A}}$ decreases remarkably. It is because higher *k* means the variance of the interference channel $h_{RA}$ is larger, thus the interference caused by R to A increases, leading to a reduction in $C_{x_{A}}$. On the other hand, varying *k* obviously does not affect $C_{x_{B}}$.

**Figure 9:**
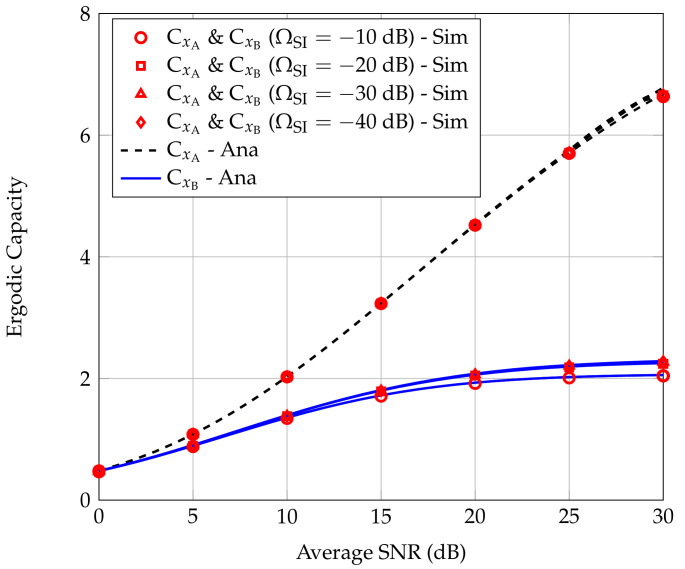
Effect of the SI cancellation coefficient on the ECs of users A and B.

Figure 9 shows the influence of the SI cancellation coefficient at R to the $C_{x_{A}}$ and $C_{x_{B}}$ in two power allocation scenarios, i.e., $\left(a_{1}=0.2,a_{2}=0.8\right)$ and $\left(a_{1}=0.3,a_{2}=0.7\right)$. The SI cancellation coefficient at R changes as −10 dB, −20 dB, −30 dB, and −40 dB. We can see that in the low SNR regime, the SI cancellation capacity in the range of −20 dB to −40 dB hardly affects $C_{x_{A}}$ and $C_{x_{B}}$, thus the value of the ECs of users tend to converge to a peak value. When $\Omega_{\mathrm{SI}}=-10$ dB, $C_{x_{B}}$ slightly attenuates in high the SNR regime, however, $C_{x_{A}}$ is almost unaffected compared to the case that $\Omega_{\mathrm{SI}}$ is in the range from −20 to −40 dB.

## 5. Conclusions

In this paper, we analyzed a downlink FD-NOMA relay system where the direct communication between the near user and the base station is possible while the communication between the far user and base station needs the support of an FD relay, which is powered wirelessly from ambient radio signals by using a power splitting protocol. We derived the exact analytical expressions of the outage probabilities and ergodic capacities at both users, then conducted Monte–Carlo simulations to validate these derived expressions. Numerical results show that when the relay harvests more energy, the possibility of successfully decoding the signal received from the base station decreases, resulting in lower OP performance and the EC at the far user. Therefore, based on the mathematical expressions in this paper, we can find an appropriate power division ratio that satisfies the quality of service requirements at the far user by using the numerical method. The application of our considered FD-NOMA relay system can be found in the case that the quality-of-service (QoS) of the far user is ensured when high-rise buildings or mountains block the link between the base station (BS) and far user. On the other hand, our considered system is suitable for applications with low data rate requirements, such as short queries and requests in IoT networks. To further improve the system performance, we can employ multiple antennas at the BS and relay.

## Figures and Tables

**Table 1:** List of main symbols and their descriptions.

Symbol	Description
$h_{\mathrm{XY}}$	Channel coefficient from X to Y, $\mathrm{XY}\;\in \;\left\{\mathrm{SA},\;\mathrm{SR},\;\mathrm{RB},\;\mathrm{RA}\right\}$
$P_{S}$	Transmission power of S
$P_{R}$	Transmission power of R
α	Power division ratio, $\alpha \;\in \;\left(0,1\right)$
ξ	Energy conversion efficiency
T	Signal transmission cycle
τ	Time delay due to FD signal processing
*k*	Strength of inter-user interference

## Appendix A. Compute *I*_1_

Substituting (23) into (22), and combining with [37] Equation (3.324.1), we can write $I_{1}$ as

(A1) $$\begin{array}{cc} I_{1} & =\int_{\frac{\sigma_{A}^{2}}{\varphi }}^{\infty }\mathrm{Pr}\left(\left(b{\left|h_{\mathrm{SR}}\right|}^{2}+c\right){\left|h_{\mathrm{RA}}\right|}^{2}<\varphi z-\sigma_{A}^{2}\right)f_{{\left|h_{\mathrm{SA}}\right|}^{2}}\left(z\right)dz\\ \; & =\int_{\frac{\sigma_{A}^{2}}{\varphi }}^{\infty }\int_{0}^{\infty }Pr\left({\left|h_{\mathrm{SR}}\right|}^{2}<\frac{\varphi z-\sigma_{A}^{2}}{by}-\frac{c}{b}\right)\;\;f_{{\left|h_{\mathrm{RA}}\right|}^{2}}\left(y\right)f_{{\left|h_{\mathrm{SA}}\right|}^{2}}\left(z\right)dz\\ \; & =\int_{\frac{\sigma_{A}^{2}}{\varphi }}^{\infty }\left(1-\frac{1}{k\lambda_{\mathrm{RA}}}\exp\left(\frac{c}{b\lambda_{\mathrm{SR}}}\right)\int_{0}^{\infty }\exp\left(-\left(\frac{\varphi z-\sigma_{A}^{2}}{b\lambda_{\mathrm{SR}}y}+\frac{y}{k\lambda_{\mathrm{RA}}}\right)\right)dy\right)\;\;f_{{\left|h_{\mathrm{SA}}\right|}^{2}}\left(z\right)dz\\ \; & =\int_{\frac{\sigma_{A}^{2}}{\varphi }}^{\infty }\left(1-\exp\left(\frac{c}{b\lambda_{\mathrm{SR}}}\right)2\sqrt{\frac{\varphi z-\sigma_{A}^{2}}{b\lambda_{\mathrm{SR}}k\lambda_{\mathrm{RA}}}}K_{1}\left(2\sqrt{\frac{\varphi z-\sigma_{A}^{2}}{b\lambda_{\mathrm{SR}}k\lambda_{\mathrm{RA}}}}\right)\right)f_{{\left|h_{\mathrm{SA}}\right|}^{2}}\left(z\right)dz, \end{array}$$

where $K_{1}\left(\cdot \right)$ denotes the first-order modified Bessel function of the second kind [37] Equation (8.432).

Applying the change of variable $t=z-\sigma_{A}^{2}/\varphi$, we obtain

(A2) $$\begin{array}{cc} \;I_{1} & =\exp\left(-\frac{\sigma_{A}^{2}}{\varphi \lambda_{\mathrm{SA}}}\right)-\frac{2}{\lambda_{\mathrm{SA}}}\sqrt{\frac{\varphi }{bk\lambda_{\mathrm{RA}}\lambda_{\mathrm{SR}}}}\exp\left(\frac{c}{b\lambda_{\mathrm{SR}}}-\frac{\sigma_{A}^{2}}{\varphi \lambda_{\mathrm{SA}}}\right)\\ \; & \times \int_{0}^{\infty }\sqrt{t}K_{1}\left(2\sqrt{\frac{\varphi }{b\lambda_{\mathrm{SR}}k\lambda_{\mathrm{RA}}}}\sqrt{t}\right)\exp\left(-\frac{t}{\lambda_{\mathrm{SA}}}\right)dt. \end{array}$$

With the help of [37] Equation (6.643.3), we have

(A3) $$\begin{array}{c} I_{1}=\exp\left(-\frac{\sigma_{A}^{2}}{\varphi \lambda_{\mathrm{SA}}}\right)-\exp\left(\frac{c}{b\lambda_{\mathrm{SR}}}-\frac{\sigma_{A}^{2}}{\varphi \lambda_{\mathrm{SA}}}+\frac{\varphi \lambda_{\mathrm{SA}}}{2b\lambda_{\mathrm{SR}}k\lambda_{\mathrm{RA}}}\right)\;\times \;W_{-1,\frac{1}{2}}\left(\frac{\varphi \lambda_{\mathrm{SA}}}{b\lambda_{\mathrm{SR}}k\lambda_{\mathrm{RA}}}\right). \end{array}$$

From (21), (22) and (A3), we obtain the exact theoretical expression of the OP at A as (17).

## Appendix B. Proof of Theorem 2

The expression (24) can be expressed as

(A4) $$\begin{array}{cc} {\mathrm{OP}}_{x_{B}} & =1-\int_{0}^{\infty }\mathrm{Pr}\left({\left|h_{\mathrm{SR}}\right|}^{2}>\frac{m\gamma_{B}}{P_{S}\left(a_{2}-a_{1}\gamma_{B}\right)},{\left|h_{\mathrm{SR}}\right|}^{2}>\frac{\gamma_{B}\sigma_{B}^{2}}{bx}-\frac{c}{b}\right)f_{{\left|h_{\mathrm{RB}}\right|}^{2}}\left(x\right)dx\\ & =1-\underset{I_{2}}{\underset{︸}{\int_{x_{0}}^{+\infty }\mathrm{Pr}\left({\left|h_{\mathrm{SR}}\right|}^{2}>\frac{m\gamma_{B}}{P_{S}\left(a_{2}-a_{1}\gamma_{B}\right)}\right)f_{{\left|h_{\mathrm{RB}}\right|}^{2}}\left(x\right)dx}}-\underset{I_{3}}{\underset{︸}{\int_{0}^{x_{0}}\mathrm{Pr}\left({\left|h_{\mathrm{SR}}\right|}^{2}>\frac{\gamma_{B}\sigma_{B}^{2}}{bx}-\frac{c}{b}\right)f_{{\left|h_{\mathrm{RB}}\right|}^{2}}\left(x\right)dx}}, \end{array}$$

where $x_{0}$ is given in (26).

Next, we calculate the first integration $I_{2}$ of (A4) as

(A5) $$\begin{array}{cc} I_{2} & =\int_{x_{0}}^{\infty }\exp\left(-\frac{m\gamma_{B}}{P_{S}\left(a_{2}-a_{1}\gamma_{B}\right)\lambda_{\mathrm{SR}}}\right)\times \frac{1}{\lambda_{\mathrm{RB}}}\exp\left(-\frac{x}{\lambda_{\mathrm{RB}}}\right)dx\\ \; & =\exp\left(-\frac{x_{0}}{\lambda_{\mathrm{RB}}}-\frac{m\gamma_{B}}{P_{S}\left(a_{2}-a_{1}\gamma_{B}\right)\lambda_{\mathrm{SR}}}\right). \end{array}$$

and the second integration $I_{3}$ of (A4) as

(A6) $$\begin{array}{cc} I_{3} & =\;\int_{0}^{x_{0}}\exp\left(-\left(\frac{\gamma_{B}\sigma_{B}^{2}}{b\lambda_{\mathrm{SR}}x}-\frac{c}{b\lambda_{\mathrm{SR}}}\right)\right)\frac{1}{\lambda_{\mathrm{RB}}}\exp\left(-\frac{x}{\lambda_{\mathrm{RB}}}\right)dx\\ \; & =\;\frac{1}{\lambda_{\mathrm{RB}}}\exp\;\left(\;\frac{c}{b\lambda_{\mathrm{SR}}}\;\right)\;\int_{0}^{x_{0}}\exp\;\left(\;-\left(\frac{\gamma_{B}\sigma_{B}^{2}}{b\lambda_{\mathrm{SR}}x}+\frac{x}{\lambda_{\mathrm{RB}}}\right)\;\right)dx. \end{array}$$

Applying Taylor series expansion for $e^{-\frac{\phi }{x}}$, i.e., $e^{-\frac{\phi }{x}}=\sum_{n=0}^{\infty }{\frac{{\left(-1\right)}^{n}}{n!}\left(\frac{\phi }{x}\right)}^{n}$, we obtain

(A7) $$\begin{array}{cc} I_{3} & =\frac{1}{\lambda_{\mathrm{RB}}}\exp\left(\frac{c}{b\lambda_{\mathrm{SR}}}\right)\times \int_{0}^{x_{0}}{\sum_{n=0}^{N}\frac{{\left(-1\right)}^{n}}{n!}\left(\frac{1}{\lambda_{\mathrm{RB}}}\right)}^{n}x^{n}\exp\left(-\frac{\gamma_{B}\sigma_{B}^{2}}{b\lambda_{\mathrm{SR}}x}\right)dx\\ \; & =\frac{1}{\lambda_{\mathrm{RB}}}\exp\left(\frac{c}{b\lambda_{\mathrm{SR}}}\right)\sum_{n=0}^{N}\frac{{\left(-1\right)}^{n}}{n!}{\left(\frac{1}{\lambda_{\mathrm{RB}}}\right)}^{n}\times \int_{0}^{x_{0}}x^{n}\exp\left(-\frac{\gamma_{B}\sigma_{B}^{2}}{b\lambda_{\mathrm{SR}}x}\right)dx. \end{array}$$

With the help of [37] Equation (3.471.2), (A7) becomes

(A8) $$\begin{array}{cc} I_{3} & =\frac{1}{\lambda_{\mathrm{RB}}}\exp\left(\frac{c}{b\lambda_{\mathrm{SR}}}\right)\\ & \times \sum_{n=0}^{N}\frac{{\left(-1\right)}^{n}}{n!}{\left(\frac{1}{\lambda_{\mathrm{RB}}}\right)}^{n}{\left(\frac{\gamma_{B}\sigma_{B}^{2}}{b\lambda_{\mathrm{SR}}}\right)}^{n/2}\;{\left(x_{0}\right)}^{1+n/2}\times \exp\left(-\frac{\gamma_{B}\sigma_{B}^{2}}{2b\lambda_{\mathrm{SR}}x_{0}}\right)W_{-1-\frac{n}{2},\frac{n+1}{2}}\left(\frac{\gamma_{B}\sigma_{B}^{2}}{b\lambda_{\mathrm{SR}}x_{0}}\right). \end{array}$$

From (A4), (A5) and (A8), we obtain the exact theoretical expression of the OP at B as (25).

## Appendix C. Proof Theorem 3

From (28), to derive the EC expression of $x_{A}$ at A, we should know the CDF of $\gamma_{x_{A}}$. To obtain this CDF, from (15), we have

(A9) $$\begin{array}{cc} F_{\gamma_{x_{A}}}\left(x\right) & =\mathrm{Pr}\left(\gamma_{x_{A}}<x\right)\;=1-\mathrm{Pr}\left(\frac{P_{S}a_{1}{\left|h_{\mathrm{SA}}\right|}^{2}}{P_{R}{\left|h_{\mathrm{RA}}\right|}^{2}+\sigma_{A}^{2}}>x\right)=1-\mathrm{Pr}\left(\frac{P_{S}a_{1}{\left|h_{\mathrm{SA}}\right|}^{2}}{\left(b{\left|h_{\mathrm{SR}}\right|}^{2}+c\right){\left|h_{\mathrm{RA}}\right|}^{2}+\sigma_{A}^{2}}>x\right)\\ & =1-\int_{\frac{\sigma_{A}^{2}}{\varphi }}^{\infty }\int_{0}^{\infty }\mathrm{Pr}\left(b{\left|h_{\mathrm{SR}}\right|}^{2}+c<\frac{\varphi z-\sigma_{A}^{2}}{y}\right)\;\times \;f_{{\left|h_{\mathrm{RA}}\right|}^{2}}\left(y\right)f_{{\left|h_{\mathrm{SA}}\right|}^{2}}\left(z\right)dydz\\ & =1-\int_{\frac{\sigma_{A}^{2}}{\varphi }}^{\infty }\int_{0}^{\frac{\varphi z-\sigma_{A}^{2}}{c}}\mathrm{Pr}\left({\left|h_{\mathrm{SR}}\right|}^{2}<\frac{\varphi z-\sigma_{A}^{2}}{by}-\frac{c}{b}\right)\;\times \;f_{{\left|h_{\mathrm{RA}}\right|}^{2}}\left(y\right)f_{{\left|h_{\mathrm{SA}}\right|}^{2}}\left(z\right)dydz. \end{array}$$

Since all wireless channels in the considered system are influenced by Rayleigh fading, we have

(A10) $$\begin{array}{c} F_{\gamma_{x_{A}}}\left(x\right)=1-\int_{\frac{\sigma_{A}^{2}}{\varphi }}^{\infty }\left(1-\exp\left(\frac{\varphi z-\sigma_{A}^{2}}{ck\lambda_{\mathrm{RA}}}\right)-I_{4}\right)f_{{\left|h_{\mathrm{SA}}\right|}^{2}}\left(z\right)dz, \end{array}$$

where

(A11) $$\begin{array}{c} I_{4}=\frac{1}{k\lambda_{\mathrm{RA}}}\exp\left(\frac{c}{b\lambda_{\mathrm{SR}}}\right)\;\times \;\int_{0}^{\frac{\varphi z-\sigma_{A}^{2}}{c}}\exp\left(-\left(\frac{\varphi z-\sigma_{A}^{2}}{by\lambda_{\mathrm{SR}}}+\frac{y}{k\lambda_{\mathrm{RA}}}\right)\right)dy. \end{array}$$

Applying Gaussian–Chebyshev quadrature approach, the integration in (A11) can be solved as

(A12) $$\begin{array}{c} I_{4}=\frac{1}{k\lambda_{\mathrm{RA}}}\exp\left(\frac{c}{b\lambda_{\mathrm{SR}}}\right)\frac{\varphi z-\sigma_{A}^{2}}{2c}\times \sum_{i=1}^{N}\omega_{N}\sqrt{1-y_{i}^{2}}\exp\left(-\left(\frac{\varphi z-\sigma_{A}^{2}}{b\lambda_{\mathrm{SR}}x_{i}}+\frac{x_{i}}{k\lambda_{\mathrm{RA}}}\right)\right), \end{array}$$

where $\omega_{N}=\pi /N$, $y_{i}=\cos\left(\frac{2i-1}{2N}\pi \right)$, $x_{i}=\frac{\varphi z-\sigma_{A}^{2}}{2c}\left(y_{i}+1\right)$, and *N* is the complexity–accuracy trade-off parameter.

Substituting (A12) into (A10) and applying the change of variables $t=z-\sigma_{A}^{2}/\varphi$, we obtain

(A13) $$\begin{array}{cc} F_{\gamma_{A}}\left(x\right) & =1-\int_{0}^{\infty }\frac{1}{\lambda_{\mathrm{SA}}}\exp\left(-\frac{\sigma_{A}^{2}}{\varphi \lambda_{\mathrm{SA}}}\right)\exp\left(-\frac{t}{\lambda_{\mathrm{SA}}}\right)dt\\ & +\int_{0}^{\infty }\frac{1}{\lambda_{\mathrm{SA}}}\exp\left(-\frac{\sigma_{A}^{2}}{\varphi \lambda_{\mathrm{SA}}}\right)\exp\left(-\left(\frac{1}{\lambda_{\mathrm{SA}}}-\frac{\varphi }{ck\lambda_{\mathrm{RA}}}\right)t\right)dt\\ & +\sum_{i=1}^{N}\frac{\omega_{N}}{k\lambda_{\mathrm{RA}}\lambda_{\mathrm{SA}}}\sqrt{1-y_{i}^{2}}\exp\left(\frac{c}{b\lambda_{\mathrm{SR}}}\right)e^{\frac{c}{b\lambda_{\mathrm{SR}}}}\\ & \times \exp\left(-\frac{2c}{b\lambda_{\mathrm{SR}}\left(y_{i}+1\right)}-\frac{\sigma_{A}^{2}}{\varphi \lambda_{\mathrm{RA}}}\right)\int_{0}^{\infty }\frac{\varphi t}{2c}\exp\left(-\left(\frac{\varphi \left(y_{i}+1\right)}{2ck\lambda_{\mathrm{RA}}}+\frac{1}{\lambda_{\mathrm{SA}}}\right)t\right)dt\\ & =1-\exp\left(-\frac{\sigma_{A}^{2}x}{P_{S}a_{1}\lambda_{\mathrm{SA}}}\right)+\frac{x}{x-\frac{P_{S}a_{1}\lambda_{\mathrm{SA}}}{ck\lambda_{\mathrm{RA}}}}\exp\left(-\frac{\sigma_{A}^{2}x}{P_{S}a_{1}\lambda_{\mathrm{SA}}}\right)\\ & +\;\Psi \frac{P_{S}a_{1}}{2c}\frac{x{\left(2ck\lambda_{\mathrm{RA}}\lambda_{\mathrm{SA}}\right)}^{2}}{{\left(P_{S}a_{1}\lambda_{\mathrm{SA}}\left(y_{i}+1\right)\;+\;2ck\lambda_{\mathrm{RA}}x\right)}^{2}}, \end{array}$$

where $\Psi \;=\;\sum_{i=1}^{N}\;\sqrt{1\;-\;y_{i}^{2}}\frac{\omega_{N}}{k\lambda_{\mathrm{RA}}\lambda_{\mathrm{SA}}}e^{\frac{c}{b\lambda_{\mathrm{SR}}}}e^{-\frac{2c}{b\lambda_{\mathrm{SR}}\left(y_{i}+1\right)}}$.

Substituting (A13) into (28), we have the EC of $x_{A}$ at A as

(A14) $$\begin{array}{cc} C_{x_{A}} & =\frac{1}{\ln2}\underset{I_{5}}{\underset{︸}{\int_{0}^{\infty }\frac{1}{x+1}\exp\left(-\frac{\sigma_{A}^{2}x}{P_{S}a_{1}\lambda_{\mathrm{SA}}}\right)dx}}-\frac{1}{\ln2}\underset{I_{6}}{\underset{︸}{\int_{0}^{\infty }\frac{1}{x+1}\frac{x\lambda_{\mathrm{SA}}}{x-P_{S}a_{1}\lambda_{\mathrm{SA}}/\left(ck\lambda_{\mathrm{RA}}\right)}\exp\left(-\frac{\sigma_{A}^{2}x}{P_{S}a_{1}\lambda_{\mathrm{SA}}}\right)dx}}\\ & -\;\Psi \frac{P_{S}a_{1}}{2c}\underset{I_{7}}{\underset{︸}{\int_{0}^{\infty }\frac{1}{x+1}\frac{x{\left(2ck\lambda_{\mathrm{RA}}\lambda_{\mathrm{SA}}\right)}^{2}}{{\left(P_{S}a_{1}\lambda_{\mathrm{SA}}\left(y_{i}+1\right)\;+\;2ck\lambda_{\mathrm{RA}}x\right)}^{2}}\exp\left(-\frac{\sigma_{A}^{2}x}{P_{S}a_{1}\lambda_{\mathrm{SA}}}\right)dx}}. \end{array}$$

After some algebraic manipulations and applying the formula ([37] Equation (3.352.4)), we obtain

(A15) $$\begin{array}{c} I_{5}=-\exp\left(\frac{\sigma_{A}^{2}}{P_{S}a_{1}\lambda_{\mathrm{SA}}}\right)\;\mathrm{Ei}\;\left(-\frac{\sigma_{A}^{2}}{P_{S}a_{1}\lambda_{\mathrm{SA}}}\right), \end{array}$$

and

(A16) $$\begin{array}{cc} I_{6} & =-\exp\left(-\frac{\sigma_{A}^{2}}{ck\lambda_{\mathrm{RA}}}\right)\;\mathrm{Ei}\;\left(\frac{\sigma_{A}^{2}}{ck\lambda_{\mathrm{RA}}}\right)\;+\frac{1}{P_{S}a_{1}\lambda_{\mathrm{SA}}/\left(ck\lambda_{\mathrm{RA}}\right)+1}\\ \; & \times \left[\exp\left(-\frac{\sigma_{A}^{2}}{ck\lambda_{\mathrm{RA}}}\right)\;\mathrm{Ei}\;\left(\frac{\sigma_{A}^{2}}{ck\lambda_{\mathrm{RA}}}\right)-\exp\left(\frac{\sigma_{A}^{2}}{P_{S}a_{1}\lambda_{\mathrm{SA}}}\right)\;\mathrm{Ei}\;\left(-\frac{\sigma_{A}^{2}}{P_{S}a_{1}\lambda_{\mathrm{SA}}}\right)\right]. \end{array}$$

Before calculating $I_{7}$, we rewrite it as

(A17) $$\begin{array}{cc} I_{7} & =\lambda_{\mathrm{SA}}^{2}\int_{0}^{\infty }\left(1-\frac{1}{x+1}\right)\frac{1}{{\left(x+\theta \right)}^{2}}\exp\left(-\frac{\sigma_{A}^{2}x}{P_{S}a_{1}\lambda_{\mathrm{SA}}}\right)dx\\ & =\lambda_{\mathrm{SA}}^{2}\int_{0}^{\infty }\frac{1}{{\left(x+\theta \right)}^{2}}\exp\left(-\frac{\sigma_{A}^{2}x}{P_{S}a_{1}\lambda_{\mathrm{SA}}}\right)dx-\lambda_{\mathrm{SA}}^{2}\;\int_{0}^{\infty }\;\left(\frac{A}{x+1}\;+\;\frac{B}{x+\theta }\;+\;\frac{C}{{\left(x+\theta \right)}^{2}}\right)\;\exp\;\left(\;-\frac{\sigma_{A}^{2}x}{P_{S}a_{1}\lambda_{\mathrm{SA}}}\;\right)dx, \end{array}$$

where $\theta =\frac{P_{S}a_{1}\lambda_{\mathrm{SA}}\left(y_{i}+1\right)}{2ck\lambda_{\mathrm{RA}}}$, $A=\frac{1}{{\left(\theta -1\right)}^{2}}$, $B=-\frac{1}{{\left(\theta -1\right)}^{2}}$, and $C=\frac{1-\theta }{{\left(\theta -1\right)}^{2}}$.

With the help of ([37] Equation (3.352.4)) and ([37] Equation (3.353.3)), we have

(A18) $$\begin{array}{cc} I_{7} & =A\exp\left(\frac{\sigma_{A}^{2}}{P_{S}a_{1}\lambda_{\mathrm{SA}}}\right)\mathrm{Ei}\left(-\frac{\sigma_{A}^{2}}{P_{S}a_{1}\lambda_{\mathrm{SA}}}\right)+B\exp\left(\frac{\theta \sigma_{A}^{2}}{P_{S}a_{1}\lambda_{\mathrm{SA}}}\right)\mathrm{Ei}\left(-\frac{\theta \sigma_{A}^{2}}{P_{S}a_{1}\lambda_{\mathrm{SA}}}\right)\\ & +\;\left(1-C\right)\lambda_{\mathrm{SA}}^{2}\times \left[\frac{\sigma_{A}^{2}}{P_{S}a_{1}\lambda_{\mathrm{SA}}}\exp\;\left(\;\frac{\theta \sigma_{A}^{2}}{P_{S}a_{1}\lambda_{\mathrm{SA}}}\;\right)\mathrm{Ei}\left(-\frac{\theta \sigma_{A}^{2}}{P_{S}a_{1}\lambda_{\mathrm{SA}}}\right)\;+\;\frac{1}{\theta }\right].\; \end{array}$$

From (A14)–(A16), and (A18), we have the EC expression of $x_{A}$ at A as (29).

## Appendix D. Proof of Theorem 4

Firstly, we find the CDF of *X*. Applying the Gaussian–Chebyshev quadrature approximation method to (A6), we obtain

(A19) $$\begin{array}{cc} I_{3} & =\frac{1}{\lambda_{\mathrm{RB}}}\exp\left(\frac{c}{b\lambda_{\mathrm{SR}}}\right)\int_{0}^{x_{0}}\exp\left(-\left(\frac{\gamma_{B}\sigma_{B}^{2}}{b\lambda_{\mathrm{SR}}x}+\frac{x}{\lambda_{\mathrm{RB}}}\right)\right)dx\\ \; & =\frac{x_{0}}{2\lambda_{\mathrm{RB}}}\exp\left(\frac{c}{b\lambda_{\mathrm{SR}}}\right)\times \sum_{i=1}^{N}\omega_{N}\sqrt{1-y_{i}^{2}}\exp\left(-\left(\frac{\gamma_{B}\sigma_{B}^{2}}{b\lambda_{\mathrm{SR}}x_{i}}+\frac{x_{i}}{\lambda_{\mathrm{RB}}}\right)\right), \end{array}$$

where $\omega_{N}=\frac{\pi }{N}$, $y_{i}=\cos\left(\frac{2i-1}{2N}\pi \right)$, $x_{i}=\frac{x_{0}}{2}\left(y_{i}+1\right)$.

From (A5) and (A19), after some manipulations, we obtain the CDF of *X* as

(A20) $$\begin{array}{cc} F_{X}\left(x\right) & =1-\exp\left(-\frac{P_{S}\sigma_{B}^{2}\left(a_{2}-a_{1}x\right)x}{mb\lambda_{\mathrm{RB}}x+cP_{S}\lambda_{\mathrm{RB}}\left(a_{2}-a_{1}x\right)}-\frac{mx}{P_{S}\left(a_{2}-a_{1}x\right)\lambda_{\mathrm{SR}}}\right)\\ & -\frac{1}{2\lambda_{\mathrm{RB}}}\frac{P_{S}\sigma^{2}\left(a_{2}-a_{1}x\right)x}{mbx+cP_{S}\left(a_{2}-a_{1}x\right)}\exp\left(\frac{c}{b\lambda_{\mathrm{SR}}}\right)\\ & \times \sum_{i=1}^{N}\omega_{N}\sqrt{1-y_{i}^{2}}\exp\left(-\left(\frac{2\left(mbx+cP_{S}\left(a_{2}-a_{1}x\right)\right)}{b\lambda_{\mathrm{SR}}P_{S}\sigma_{B}^{2}\left(y_{i}+1\right)\left(a_{2}-a_{1}x\right)x}+\frac{1}{2}\frac{P_{S}\sigma_{B}^{2}\left(y_{i}+1\right)\left(a_{2}-a_{1}x\right)x}{mb\lambda_{\mathrm{RB}}x+cP_{S}\lambda_{\mathrm{RB}}\left(a_{2}-a_{1}x\right)}\right)\right). \end{array}$$

Substituting (A20) into (32) with notice that $\left(a_{2}-a_{1}x\right)>0$ leads to $0<x<u=a_{2}/a_{1}$, we get

(A21) $$\begin{array}{cc} C_{x_{B}} & =\frac{1}{\ln2}\int_{0}^{u}\frac{1}{1+x}\exp\left(-\frac{P_{S}\sigma_{B}^{2}\left(a_{2}-a_{1}x\right)x}{mb\lambda_{\mathrm{RB}}x+cP_{S}\lambda_{\mathrm{RB}}\left(a_{2}-a_{1}x\right)}-\frac{mx}{P_{S}\left(a_{2}-a_{1}x\right)\lambda_{\mathrm{SR}}}\right)dx\\ \; & +\frac{1}{2\ln2}\frac{1}{\lambda_{\mathrm{RB}}}\exp\left(\frac{c}{b\lambda_{\mathrm{SR}}}\right)\sum_{i=1}^{N}\omega_{N}\sqrt{1-y_{i}^{2}}\\ \; & \times \;\;\int_{0}^{u}\frac{1}{\;1\;+\;x}\frac{P_{S}\sigma_{B}^{2}\left(\;a_{2}\;-\;a_{1}x\right)x}{mbx\;+\;cP_{S}\left(\;a_{2}\;-\;a_{1}x\right)}\;\exp\left(\;-\;\left(\;\frac{2\left(mbx+cP_{S}\left(a_{2}-a_{1}x\right)\right)}{b\lambda_{\mathrm{SR}}P_{S}\left(y_{i}+1\right)\left(\;a_{2}\;-\;a_{1}x\right)}+\frac{1}{2}\frac{P_{S}\sigma_{B}^{2}\left(y_{i}+1\right)\left(\;a_{2}\;-\;a_{1}x\right)x}{mb\lambda_{\mathrm{RB}}x\;+\;cP_{S}\lambda_{\mathrm{RB}}\left(\;a_{2}-\;a_{1}x\right)}\;\right)\;\right)dx. \end{array}$$

Applying Gaussian–Chebyshev quadrature approach, we have the approximate expression of the EC of $x_{B}$ at B as (34), where $\omega_{K}=\frac{\pi }{K}$, $y_{k}=\cos\left(\frac{2k-1}{2K}\pi \right)$ and $x_{k}=\frac{u}{2}\left(y_{k}+1\right)$.
